# Effective Removal of Cyanide and Heavy Metals from an Industrial Electroplating Stream Using Calcium Alginate Hydrogels

**DOI:** 10.3390/molecules25215183

**Published:** 2020-11-07

**Authors:** Benita Pérez-Cid, Sergio Calvar, Ana Belén Moldes, Jose Manuel Cruz

**Affiliations:** 1 Department of Analytical and Food Chemistry, Faculty of Chemistry, University of Vigo, As Lagoas-Marcosende s/n, 36310 Vigo, Spain; benita@uvigo.es; 2 Department of Chemical Engineering, School of Industrial Engineering, University of Vigo, As Lagoas-Marcosende s/n, 36310 Vigo, Spain; sergiocangas@gmail.com (S.C.); jmcruz@uvigo.es (J.M.C.)

**Keywords:** cyanide, heavy metals, electroplating wastewater, alginate beads, kinetic sorption

## Abstract

A real electroplating wastewater, containing 51,190 mg/L of free cyanide (CN_f_), 4899 mg/L of Ni and 1904 mg/L of Cu, was treated with calcium alginate hydrogel beads (CAB), pure or impregnated with biodegraded grape marc (EBGM) or activated carbon (EAC) in order to reduce the elevated load of toxic pollutants below the regulatory limits. It was evaluated the effect of increasing the amount of bioadsorbent as well as the influence of two successive adsorption cycles in the removal efficiency of pollutants. The most favourable sorption conditions onto CAB provided removal percentages of 85.02% for CN_f_ and between 93.40–98.21% for heavy metals regarding the raw wastewater. The adsorption capacity of each pollutant onto CAB was considerably increased during the first 30 min of contact time, but after achieving the equilibrium, the following sorption capacities were obtained: 1177, 107.3, 39.5 and 1.52 mg/g for CN_f_, Ni, Cu and Zn, respectively. The kinetic adsorption of pollutants onto the CAB was adjusted to different kinetic models, observing that kinetic data agreed with the pseudo-second-order model. The information about intraparticle diffusion mechanisms in the bioadsorption process was also interpreted.

## 1. Introduction

Cyanide is cytotoxic for animals and humans, inhibiting the cytochrome oxidase enzyme and preventing the transport of oxygen into the cells [1]. Additionally, chronic exposure of humans and other living organisms to cyanide causes damage of central nervous system with cardiovascular and respiratory disorders as well as endocrine malfunctioning [2]. Cyanide is widely used in electroplating industries in order to assure the high quality of the finished products and, consequently, is a common contaminant in different industrial effluents including metal processing, gold mining, and plastics [3] which generally co-exists with other toxic substance such as heavy metals and degreasing solvents. Cyanide, in wastewater, mainly exists in different chemical forms: Free cyanide and associated with metallic elements forming cyanide complexes. Free cyanide (CN_f_) includes the most toxic forms: Cyanide anion (CN^−^) and hydrogen cyanide (HCN). However, cyanide complexes are classified according to the stability of the metal cyanide bond. Weak and moderately strong cyanide-complexes refer to complexes such as Cd, Cu, Ni and Zn and strong cyanide-complexes include complexes such as Fe, Au and Co [4]. 

Industrial effluents generally contain values lower than 10 mg/L of total cyanide; however, some effluents from electroplating plants and other metal finishing processes may contain considerably higher values (10,000–30,000 mg/L) and even can reach 100,000 mg/L [5]. These highly contaminated effluents must be adequately treated before to be discharged, being the contents of cyanide in wastewater serves limited by most of the countries, based on the values stipulated by their respective environmental protection agencies. The US Environmental Protection Agency (USEPA) has proposed a limit of 1.0 mg/L of total cyanide for effluents from electroplating industries of common metals that discharging ≥38.0 L per day to public owned treatment works [6] Galician regulations also have the maximum allowable values of free cyanide (0.5 mg/L) and total cyanide (1.0 mg/L) for limited discharges into the public wastewater treatment service [7]. In view of these considerations, cyanide removal from industrial effluents is necessary in order to reduce its content below the regulatory limits.

Diverse technologies have been employed for cyanide removal including alkaline chlorination [8], hydrogen peroxide oxidation [9], precipitation [10,11], adsorption [12], biological degradation [13], reverse osmosis [14], and electrodialyisis [15]. A great part of these methods can be affected by important disadvantages like elevated cost with special equipment and maintenance, low removal efficiency, sensitive operation conditions, and generation of large amounts of wastes. Therefore, adsorption methods can be considered a potentially preferred alternative for the removal of cyanide from wastewater given that its flexibility of design, simple to operate, reduced cost, no added hazardous chemicals and even the adsorbent can be significantly regenerated. 

Commercial or synthetic adsorbents such as activated carbon [16] or sulphur impregnated activated carbon [17], zero-valent iron [18], or nitrate intercalated Ni-Al layered double hydroxide [19] have been employed for adsorption of cyanide. Different biodsorbents prepared from agro-industrial solid wastes such as rice husk [20], pistachio hull waste [21], and acid treated eggshells [22] have also been used in this way. It is important to indicate that same bio-wastes and other similar materials like grape stalks [23] or banana peels [24] can also be suitable for the adsorption of heavy metals from polluted wastewater [25]. On the other hand, calcium alginate is a natural biopolymer extracted from marine algae that has been widely employed as a green and low cost bioadsorbent for effective remediation of different contaminants from wastewater, including heavy metals [26,27]. This biopolymer has been impregnated with different materials, including bio-wastes, to enhance their sorption capacities. Diverse lignocellulosic residues such as biodegraded grape mar), vineyard pruning wastes, and peat have been entrapped in calcium alginate hydrogel beads (CAB) in order to eliminate Cu from water [28], dye compounds from winery wastewater [29] and anionic-cationic micronutrients from agro-industrial effluents [30]. In a recent application, the alginate biopolymer was used for efficient removal of total organic acid anions and heavy metal ions from industrial lean methyldiethanolamine solvent used in the natural gas industry [31]. 

Moreover, other natural polymers based on chitosan [32,33] and microbial biomass [34] have also evaluated to remove hazardous substances form wastewater. However, no works were found in the literature focused on cyanide removal from industrial effluents and, hence it could be an interesting application given the acute toxicity of cyanide and the great adsorption versatility of the alginate biopolymer.

By taking the above into account, the main aims of this work were the following: (i) Determine the contents of main pollutants in an industrial wastewater from a nickel electroplating factory and estimate its potential dangerousness according current regulations; (ii) evaluate the removal efficiency of calcium alginate hydrogel beads (CAB), entrapped biodegraded grape marc (EBGM) or entrapped activated carbon (EAC) in CAB for the simultaneous removal of free cyanide and other co-existing heavy metals (Ni, Cu and Zn) from the raw wastewater; (iii) evaluate the influence of two successive adsorption cycles using CAB in the removal efficiency of all pollutants studied, regarding both the raw wastewater and the already treated water after the first treatment step; (iv) evaluate the effect of the contact time in the adsorption capacity of each pollutant onto CAB and (v) evaluate the kinetic adsorption behaviour of each pollutant onto CAB.

## 2. Results and Discussion

### 2.1. Composition of the Raw Wastewater 

The wastewater analysed in this work was collected from a plating bath from a nickel electroplating industry and several pollutants (CN_f_, Ni, Cu, Zn, Pb, Cr (VI), Cr total) were quantified in order to determine its initial composition. The results obtained and the pH value (12.45) are shown in Table 1 and all of them are expressed as mean value and standard deviation of three determinations. It is important to highlight the elevated content of CN_f_ quantified (51,190 mg/L), which is in good agreement with other plating baths previously analysed with values of 30,000 mg/L of CN_Total_ [13] or even ranging from 45,000–100,000 mg/L of CN_Total_ [5]. However, considerably lower values of CN_Total_ (76 or 94.1 mg/L) and CN_f_ (47.9 mg/L) were found in previous real electroplating wastewaters [1,22]. Other industrial effluents from coke plants (25.7 mg/g) or chemical industry (10.4–50.9 mg/g) also have more reduced contents of CN_Total_ [13]. In addition, the contents of Ni (4899 mg/L) and Cu (1904 mg/L) obtained were significantly higher than those found in other industrial plating wastewaters, where the values of Ni and Cu were, respectively, always lower than 271 and 36.16 mg/L [11,35]. Whereas Zn values (58.5 mg/L) found in this raw wastewater were relatively similar [11] or even lower than those found in this kind of industrial effluents [35]. Other heavy metals like Pb, Cr total and Cr (VI) were not detected by means of the analytical methodology employed. Maximum allowable values considered by the US Environmental Protection Agency (USEPA) for effluents from electroplating industries of common metals that discharge ≥38.0 L per day to public owned treatment works [6] and by the Galician regulations for limited discharges into the public wastewater treatment service [7] are included in Table 1. These limit values are 0.5 mg/L for CN_f_ and 1 mg/L for CN_Total_ and, in the case of heavy metals (Ni, Cu and Zn), the limit values ranged from 2–3 mg/L depending on the element. It means that real wastewater studied can be considered highly dangerous and, consequently, its load of pollutants must be considerably reduced prior to be discharged, in fact the company has to pay a high cost for the treatment of this wastewater by an external company dedicated to the treatment of industrial wastewater. 

### 2.2. Optima Bioadsorption of Pollutants

**Figure 1:**
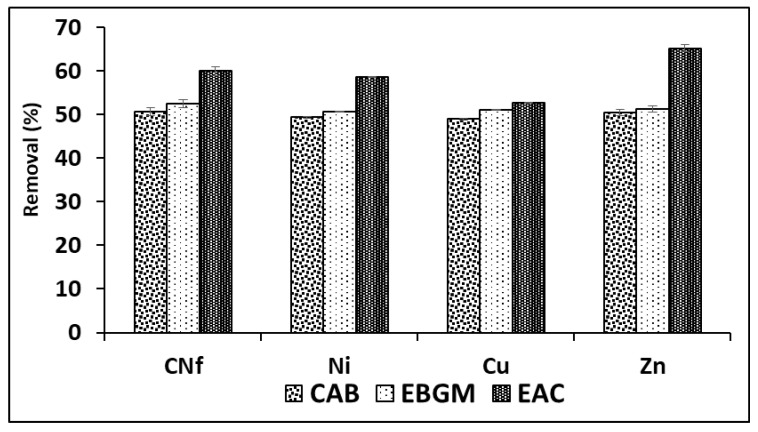
Percentages of removal of free cyanide and heavy metals onto three different bioadsorbents (CAB, EBGM and EAC) using a ratio 1:1 (*v*/*v*) between bioadsorbent/raw wastewater.

In order to obtain the best adsorption conditions for all pollutants studied different adsorbents were employed consisting of CAB (calcium alginate hydrogel), EBGM (calcium alginate hydrogel containing biodegraded grape marc) or EAC (calcium alginate hydrogel containing activated carbon), at different ratios between bioadsorbent/raw wastewater. In Figure 1 are shown the percentages of removal obtained for all pollutants using the three bioadsorbents tested (CAB, EBGM and EAC), using always a ratio 1:1 (*v/v*). As can be observed, the removal efficiencies obtained with CAB and EBGM were very similar for all pollutants with values ranged from 49.0–50.6 and 50.7–52.6%, respectively. In fact, the results obtained using both CAB and EBGM were statistically comparable (*t*-test) for each pollutant and the calculated *t*_value_ and *t*_critical_ are shown in Table 2, where is observed that for CN_f_ and Zn were not found significant differences (*p* = 0.05) between the results obtained using the two compared bioadsorbents; in contrast, for Ni and Cu the *t*_value_ is always higher than *t*_critical_ for both 95 and 99% of probability and, consequently, the results compared were significantly different. It means that EBGM provides slightly higher removal efficiencies for these two elements, with differences of only 1.3% for Ni and 2.1% for Cu. 

The results of ANOVA analysis are also shown in Table 2, where is observed that all calculated F_value_ are always higher than F_critical_ for known degrees of freedom (F_critical_ = 5.143 in this case). In addition, *p* values were equal zero in all cases indicating that, for any level of significance, the results compared were statistically different for all pollutants studied. It means that the adsorption capacities of the three bioadsorbents tested were significantly different the highest removal efficiencies (52.7–65.2%) when using EAC as bioadsorbent. The increase on removal efficiency of EAC in comparison with CAB was only 3.6% for Cu, about 9.3% for CN_f_ and Ni and 14.7% for Zn, being the last element the least abundant in the industrial wastewater studied. According to the above, it is possible to confirm that the incorporation of commercial activated carbon did not increase, in high extend, the adsorption capacity of CAB. The next experiments were carried out with CAB compose only by calcium alginate, avoiding the use of non-ecofriendly activated carbon and reducing costs. Therefore, CAB in absence of commercial activated carbon reached acceptable removal efficiencies for all pollutants (49.0–50.6%) when using a ratio bioadsorbent/raw water 1:1 (*v*/*v*). 

**Figure 2:**
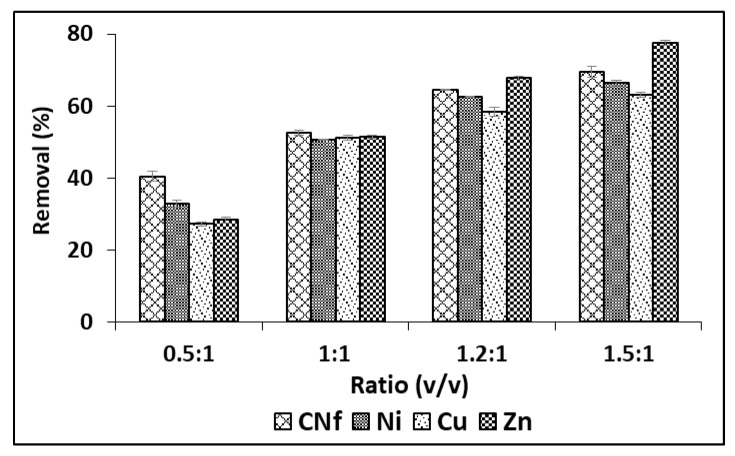
Percentages of removal of free cyanide and heavy metals onto CAB using different ratios between bioadsorbent/raw wastewater.

Although in order to enhance these adsorption capacities, different ratios were studied from 0.5:1 to 1.5: 1 (*v*/*v*) and the results are shown in Figure 2, observing that the adsorption capacity of all pollutants was considerably enhanced when the amount of bioadsorbent was increased, reaching the highest percentages of removal with ratios 1.5:1 (*v*/*v*). Thus, under these conditions Cu, Ni and CN_f_ were removed from 63.2 to 69.5% and this value was increased until 77.6% for Zn. The corresponding adsorption capacities were 1177, 107.3, 39.5 and 1.52 mg/g for CN_f_, Ni, Cu and Zn, respectively.

In previous works were found higher adsorption capacities for Cu (87.4–90.7 mg/g) and similar values for Ni (49.2 mg/g) when using calcium alginate hydrogel beads for metal sorption [26,27], but the charge and the amount of pollutants in the initial wastewater was lower than that evaluated in the current work. Other authors have used calcium alginate hydrogel beads impregnated with biodegraded grape residue to eliminate Cu from aqueous solutions and they have found considerably higher capacity values (2785 mg/g) when using the optima sorption conditions [28]. It is important indicate that the less-favourable adsorption capacities obtained in the current work for heavy metals onto CAB can be attributed to a high load of several pollutants in the real wastewater analysed, mainly cyanide, which can compete by the active sites of the bioadsorbent, which can be saturated faster. 

In the case of cyanide, no reference data were found in the literature regarding its elimination using alginate hydrogel beads, however the maximum adsorption capacity of cyanide using different commercial or synthetic adsorbents such as sulphur impregnated in activated carbon (126.77 mg/g) [17], zero-valent iron (277.77 mg/g) [18] and nitrate intercalated Ni-Al layered double hydroxide (167 mg/g) [19] were significantly lower. Several agroindustrial by-products were also used for cyanide removal and their adsorption capacities were 156.2 mg/g when using pistachio hull waste [21] and even lower values when using acid treated eggshells (126.6 mg/g) [22] or rice husk (0.40 mg/g) [20].

The structure of the bioadsorbent under study have been already studied in previous works related with the removal of pollutants from a winery wastewater [36,37]. However, they were included macro view images of the bioadsorbent before and after treatment of the electroplating wastewater under study as well as scanning electron microscope images (SEM) in the graphical abstract.

### 2.3. Effect of Two Successive Bioadsorption Cycles

The wastewater obtained after the first treatment cycle still contains a high load of contaminants and does not meet the requirements established by current regulations [6,7] to be discharged into the public treatment systems. With the aim to reduce its pollutant load, pre-treated wastewater was treated again with new CAB and using two different ratios 0.5:1 and 1:1 (*v*/*v*). The results of these experiments are shown in Table 3 and all of them are expressed as mean value and standard deviation of three determinations. It is important to indicate that although the first cycle of treatment was greatly effective, still remains high content of pollutants giving a pre-treated wastewater with 15,659, 1641, 702 and 13.2 mg/L for CN_f_, Ni, Cu and Zn, respectively. 

As it was above indicated, the second cycle of treatment was developed using two ratios CAB/wastewater, 0.5:1 and 1:1 (*v*/*v*) and, in both cases, the percentages of removal were calculated regarding pre-treated water, after the first cycle, and also regarding the raw wastewater. As it can be observed in data of Table 3, Ni and Cu present a similar behaviour, being reduced its contents between 92.52–93.59% (ratio 0.5:1) and around 94% (ratio 1:1) regarding the pre-treated wastewater. When the removal efficiency of these two metals was calculated based on the composition of raw wastewater, they reached percentages of removal between 97.25–97.85% (ratio 0.5:1) and between 97.97–98.21 (ratio 1:1). Although the results obtained for Ni and Cu are relatively similar, when the percentages of removal obtained with the two ratios, regarding both the first-cycle treated water, namely pre-treated wastewater, and the initial raw wastewater, were statistically compared (*t*-test, *p* = 0.05) significant differences were found between them. Therefore, the ratio 1:1 (*v*/*v*) provides the best results, although the increase in removal efficiency was lower than 0.72% for raw wastewater, and between 1.08–1.96% for pre-treated wastewater. CN_f_ and Zn also present a similar behaviour after the second cycle of treatment. Comparing CN_f_ and Zn, the adsorption of CN_f_ reached lower percentage removal values, what can be attributed to the elevated content of this pollutant (15,659 m/L) in the pre-treated water respect to Zn (13.2 mg/L), giving removal percentages for CN_f_ of 30.31% and 54.44% for Zn at ratio 0.5:1 *v*/*v*, increasing these values to 51.04% for CN_f_ and 70.46 for Zn at ratio 1:1 *v*/*v*. The removal efficiencies calculated respect to the raw wastewater were enhanced 6.34% for CN_f_ and 3.59% for Zn when using the ratio 1:1 (*v*/*v*), thus providing the best removal results. 

Finally, the most favourable conditions for the second cycle of treatment (ratio 1:1 *v*/*v*) provided global removal efficiencies of 85.02 for CN_f_, 93.40% for Zn and between 97.97–98.21% for Cu and Ni regarding the raw wastewater highly polluted. 

### 2.4. Kinetic Studies

#### 2.4.1. Influence of Contact Time on Bioadsorption

**Figure 3:**
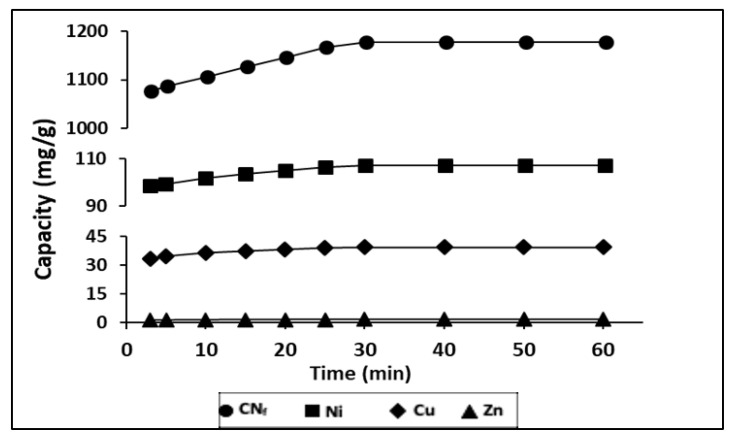
Influence of contact time in the adsorption capacity of all pollutants studied onto CAB.

During a time ranged from 3–60 min it was evaluated the influence of contact time on the adsorption capacity of heavy metals, contained in the electroplating wastewater, onto CAB, with a ratio bioadsorbent/wastewater 1.5:1 (*v*/*v*). The results obtained are shown in Figure 3, observing that between 3 and 10 min exist, a rapid adsorption of all pollutants, indicating a high affinity by the bioadsorbent. 

In fact, with only 3 min of contact were achieved adsorption capacities of 1077, 98.8, 33.5 and 1.24 mg/g for CN_f_, Ni, Cu and Zn, respectively. During the first 30 min of contact time these values were increased until 1177, 107.3, 39.5 and 1.52 mg/g, respectively. These values remain constant until 60 min, which may be attributed to the saturation of the all surface binding sites. The same behaviour was also observed for Ni, Zn and Pb removed onto calcium alginate hydrogel beads, where a rapid metal ion uptake takes place in the first 40 min and the equilibrium was achieved after 90 min of contact time [38]. In previous work, it was also evaluated the influence of the contact time on the sorption capacity of Cu and Ni onto calcium alginate hydrogel beads and it was observed a rapid sorption during the first 30 min for Ni and during the first 80 min for Cu, reaching the saturation values after 90 min [27]. In the case of cyanide there are no available results of sorption onto CAB, however other authors have evaluated its removal from wastewater by adsorption onto pistachio hull wastes and they have also observed that the rate of cyanide uptake was higher within first 30 min of contact time [21] which is in good agreement with the result reported in this work. 

#### 2.4.2. Kinetic Models 

**Figure 4:**
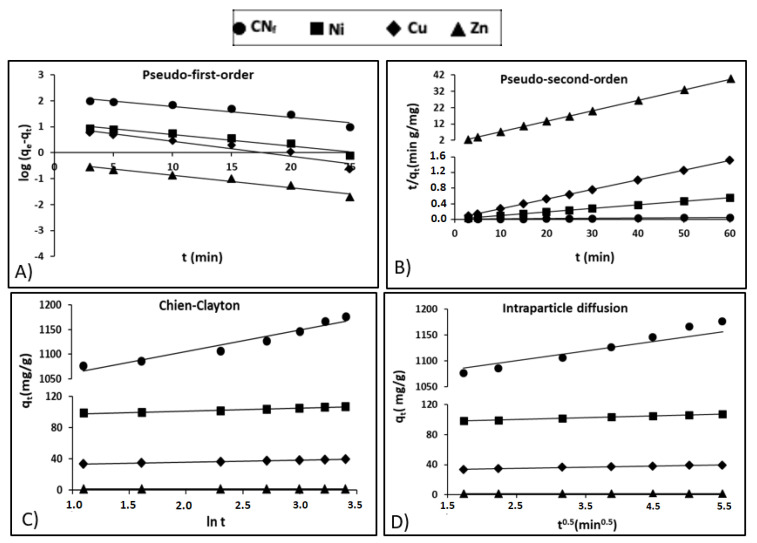
Kinetic plots for the adsorption of free cyanide and heavy metals onto CAB: (**A**) Pseudo-first-order; (**B**) Pseudo-second-order; (**C**) Chien-Clayton; (**D**) Intraparticle diffusion.

Lagergren, et al. [39] obtained a pseudo-first-order kinetic model (Equation (1)), to explain the kinetic of adsorption process:

(1) $$\log\;(q_{e}-q_{t})=\log\;q_{e}-\;\frac{k_{1}}{2.303}\;t\;$$

where *q*_e_ (mg/g) is the adsorption capacity at the equilibrium, *q*_t_ (mg/g) is the capacity at any time *t* and *k*_1_ (1/min) is a rate constant. Figure 4A shows the pseudo-first-order model adjustment of experimental data, with a relative high coefficient of determination for Zn (r^2^ = 0.961) and lower values for CN_f_, Ni and Cu (r^2^ between 0.910 and 0.935), what exhibits a deficient fit of the pseudo-first-order model, particularly to the last three pollutants. In Table 4 are shown the kinetic parameters obtained for all pollutants studied, observing important differences between the theoretical capacities estimated by the pseudo-first-order kinetic model (160.1, 13.83, 10.44 and 0.415 mg/g for CN_f_, Ni, Cu and Zn, respectively) and the experimental values: 1177, 107.3, 39.5 and 1.52 mg/g for CN_f_, Ni, Cu and Zn, respectively, therefore this model was discarded to explain the adsorption process. 

Following it is included Equation (2), that describes the adsorption kinetics using the pseudo-second-order established by Ho and McKay [40]:

(2) $$\frac{t}{q_{t}}=\;\frac{1}{k_{2}q_{e}^{2}}+\;\frac{1}{q_{e}}\;t\;$$

where, the rate constant at the equilibrium for the pseudo-second-order kinetic adsorption is defined as *k*_2_ (g/mg min). Figure 4B shows the adjustment of experimental data to pseudo-second-order kinetic model, observing determination coefficients (r^2^) close to 1.00 for all pollutants. Table 4 shows the experimental and theoretical capacities predicted by the model with differences lower than 1.4 mg/g for Ni, Cu and Zn, whereas in the case of CN_f_ the experimental sorption capacity was 73 mg/g lower than that estimated by the model. Nevertheless, it is important to remark, that CN_f_ is the pollutant that present the higher affinity by the bioadsorbent, reaching the most favourable experimental adsorption capacity (1177 mg/g), followed by Ni (107.3 mg/g), Cu (39.5 mg/g) and Zn (1.52 mg/g). In contrast, the values of equilibrium rate constant (*k*_2_) followed the reverse order, the highest corresponding to Zn (0.6508 g/mg min) and the lowest to CN_f_ (0.00128 g/mg min). In previous work focused on the cyanide adsorption onto pistachio hull wastes was proved that the values of equilibrium rate constant (*k*_2_) decrease when increase the cyanide concentration in the water stream, reaching *k*_2_ values of 0.30 g/mg min, for cyanide concentrations of 50 mg/L, that decrease to 0.028 g/mg min for cyanide concentrations of 200 mg/L. It may be attributed to the enhanced mass transfer rate with an increased concentration gradient [21]. Although the available information about cyanide adsorption is very limited, some authors [21,22] have found that pseudo-second-order model was the model that better described the adsorption of cyanide onto pistachio hull wastes and acid treated eggshells, respectively. In the case of heavy metals, a previous study also confirms that the pseudo-second-order kinetic model was the model that better adjusted the adsorption of Ni, Zn and Pb onto calcium alginate hydrogel beads [38]. In this study, the initial concentration of metals in the wastewater was 60 mg/L and the values of equilibrium rate constant (*k*_2_) obtained for both Ni (0.006 g/mg min) and Zn (0.012 g/mg min) were considerably lower than those found in the current work.

Additionally, it was evaluated the kinetic model based on the Elovich equation (Equation (3), which was simplified by Chien and Clayton [41], obtaining Equation (4).

(3) $$\frac{dq_{t}}{dt}=\;\alpha \exp\;\left(-\beta q_{t}\right)\;$$

Additionally, Equation (3) can be simplified, obtaining Equation (4) [42] by assuming *αβt* ≫ 1, and considering the boundary conditions *q*_t_ = 0 for *t* = 0 and *q*_t_ = *q*_t_ at *t* = *t*, where the initial sorption rate is defined as α (min mg/g) whereas the extent of surface coverage and activation energy for chemisorption is defined as β (g/mg).

(4) $$q_{t}=\frac{1}{\beta }\;(\ln\left(\alpha \beta \right)+\frac{1}{\beta }\ln t)\;$$

The adjustment of experimental results to the Chien-Clayton expression was shown in Figure 4C, reaching high determination coefficients for Cu and Zn (r^2^ = 0.996), followed by Ni (r^2^ = 0.963) and CN_f_ (r^2^ = 0.942). Regarding the initial adsorption rate Table 4 show favourable α values for CN_f_ (5.43 × 10^11^) and Ni (1.58 × 10^11^), followed by Cu (2.66 × 10^5^) and considerably lower for Zn (1247.3). This is in consonance with the more favourable capacities obtained for CN_f_ (1077 mg/g) and Ni (98.8 mg/g) with only 3 min of contact time, whereas for Cu (33.5 mg/g) and specially for Zn (1.24 mg/g) these values were considerably more reduced. No available information was found in the literature about the application of Chien-Clayton model to kinetic sorption studies of cyanide or heavy metals onto CAB or other sorbents and, consequently, the α values predicted by the model in this work cannot be compared. 

Moreover, Weber and Morris [42] proposed the intraparticle diffusion model (Equation (5)) which was also evaluated:

(5) $$q_{t}=k_{p}t^{0.5}+C$$

where *k*_p_ is the intraparticle diffusion rate constant (mg/g min^0.5^) and *C* is the intercept (mg/g). *C* can possess values equal or different to 0, when *C* = 0 is indicative of pure intraparticle diffusion; whereas when *C* is different from 0, the liquid fill diffusion around the particles is involved in the adsorption process. In Table 4, are included the kinetic parameters obtained for all pollutants (values from 1.13 for Zn to 1024.9 for CN_f_) and, therefore, it is assumed that film diffusion also played a role in the adsorption onto CAB, especially for CN_f_. In addition, in Figure 4D it is observed a good correlation between experimental and theoretical data for this model, with high determination coefficients for both CN_f_ and Ni (r^2^ = 0.990–0.996) followed by Cu and Zn (r^2^ = 0.983–0.987). The values of intraparticle diffusion rate constant (*k*_p_) were considerably more elevated for CN_f_ (27.487) than for heavy metals (*k*_p_ = 2.385–0.0727), what suggest that cyanide ions diffuse more quickly than heavy metals among the particles of the adsorbent. In a previous work was proved that *k*_p_ values for cyanide ions increased from 0.614 to 1.627 when increasing the concentration of CN_f_ ions in the water stream from 50 to 200 mg/L, which reconfirms the enhancement of adsorption capacity when increase the cyanide concentration [21]. In the case of heavy metals, in a previous work was suggest that heavy metal ions like Ni, Zn and even Pb diffuse quickly among the particles of CAB, particularly at the beginning of the adsorption process. The *k*_p_ values obtained were 4.91 for Zn and 3.73 for Ni, being in good agreement with the results obtained in the current work, especially in the case of Ni [38].

## 3. Materials and Methods

### 3.1. Industrial Wastewater

The analysed raw wastewater was collected from a nickel electroplating located next to the Vigo town (Galicia, North-West Spain). The sample was stored at 4 °C until use. pH, CN_f_ and diverse heavy metals like Ni, Cu, Zn, Pb, Cr (VI) and Cr total were determined in the initial sample for its characterization.

### 3.2. Preparation of Bioadsorbents 

The adsorbents used in this work consisted of calcium alginate hydrogel beads (CAB), entrapped biodegraded grape mar (EBGM) or entrapped activated carbon (EAC) in calcium alginate hydrogel beads. Calcium alginate hydrogel beads (CAB) were prepared by dissolving 2% (*m*/*v*) calcium alginate (from Panreac) in warm water, forming an emulsion that was pumped into a solution of calcium chloride (from Acros Organics), with a flow rate of 0.58 mol/L [28]. 

Entrapped biodegraded grape marc (EBGM) or entrapped activated carbon (EAC) were obtained similarly to CAB but adding to the emulsion containing calcium alginate 2% of powder biodegraded grape marc or 2% of powder activated carbon (supplied from Panreac). It is important to notice that biodegraded grape marc was obtained by spontaneous biodegradation a room temperature, during 2 months following the methodology proposed in previous works [43].

Regarding the physical properties of the bioadsorbents used in this work, these were characterized in previous works [36,37], observing that calcium alginate beads without the addition of lignocellulosic residues possess an area about 7.88–8.16 mm^2^, perimeter about 13.05–12.12 mm, roundness 0.61–0.77 μm and compactness of 0.91–0.96 μm. This range of physical parameters were obtained for calcium alginate beads formulated at concentrations of sodium alginate around 2% and concentrations of calcium chloride between 0.05 and 0.9 mol/L, observing that the calcium alginate concentration used in the formulation did not affect the physical properties of calcium alginate beads as it was added in excess to the formulation [37].

On the other hand, the inclusion of lignocellulosic adsorbents in the calcium alginate beads increases the roundness of calcium alginate beads but did not affect in high extent the other physical parameters evaluated. Following are included the physical properties studied in a previous for calcium alginate beads containing biodegraded grape marc: Area 7.66 mm^2^, perimeter 11.02 mm, roundness 0.79 μm and compactness of 0.94 μm [37].

### 3.3. Bioadsorption of Pollutants

Adsorption experiments were carried out in order to evaluate the influence of EBGM, EAC and CAB in the adsorption capacity of each pollutant, contained in the industrial electroplating stream, as well as the effect of increasing the amount of bioadsorbent and the influence of two successive adsorption cycles in the pollutants removal efficiency achieved. Three different experiments were developed consisting of: (i) Adsorption processes treating 10 mL of raw wastewater with three different bioadsorbents (CAB, EBGM and EAC), using always a ratio 1:1 (*v*/*v*) between bioadorbent/raw wastewater; (ii) adsorption processes treating 10 mL of raw wastewater with CAB using different ratios, ranged from 0.5:1 to 1.5:1 (*v*/*v*); (iii) adsorption processes developed in two successive treatment cycles, in the first cycle were treated 10 mL of raw wastewater with CAB using a ratio 1.5:1 (*v*/*v*) and in the second cycle, the wastewater from the first cycle was again treated with new CAB using two different ratios 0.5:1 and 1:1 (*v*/*v*). Batch adsorption assays were carried out by mixing the bioadorbent and the raw wastewater in 100 mL Erlenmeyer flaks, with a contact time of 30 min in a shaker at 120 rpm and room temperature. After the adsorption process, the biodasorbent was removed and the electroplating wastewater was filtered using syringe filters of 0.45 µm. The final solution was stored at 4 °C until analysis.

### 3.4. Analysis of Pollutants 

The heavy metals contained in the electroplating, wastewater, before and after treatment, were determined by Flame Atomic Absorption Spectrometry (Thermo Scientific, iCE 3000 series, Waltham, MA, USA) using air-acetylene flame and hollow cathode lamps as radiation source. The instrumental parameters were chosen for each element according to the manufacturer recommendations. The stock solutions of metals (1000 ± 2 mg/L) were supplied by Panreac. The working standard solutions, for each element, were daily prepared by adequate dilution the stock solution. Hexavalent chromium was determined by reaction with 1,5-diphenylcarbazide in acid solution [44]. A red-violet coloured complex was formed, which was quantified at 540 nm using an UV-Spectrophotometer (Varian, Cary 100 model, Santa Clara, CA, USA). Potassium dichromate and 1,5-diphenylcarbazide reagents were supplied by Merck. Free cyanide (CN_f_) was quantified by means of the Tritimetric Standard Method 4500-CN^−^ (D) using silver nitrate as standard titrant and *p*-dimethylaminobenzal-rhodanine as indicator solution [45]. The two last reagents were both supplied by Sigma Aldrich.

The adsorption capacity (*q*) and removal efficiency (*R*) for each pollutant was calculated using Equations (6) and (7), respectively.

(6) $$q\;(mg/g)=\frac{\left(C_{0}-C_{t}\right)\cdot V}{W}$$

(7) $$R\;\left(\%\right)=\frac{\left(C_{0}-C_{t}\right)}{C_{0}}\cdot 100$$

where *C*_0_ and *C_t_* are initial and final concentrations of each pollutant (mg/L) respectively; *V* is volume of the aqueous solution (L) and *W* is the mass of bioadsorbent (g).

### 3.5. Kinetic Studies

The kinetic study involves specific experiments in order to evaluate the effect of adsorption time in the capacity of all the pollutants studied. The kinetic experiments were developed using the procedure described in Section 3.3, although using only CAB as bioadsorbent with a ratio bioadsorbent/raw wastewater 1.5:1 (*v*/*v*). Kinetic experiments were carried out during 3–60 min and, at different intervals of time, samples of electroplating wastewater were obtained in order to evaluate the adsorption capacity of CAB. It is important to remark that individual experiments were developed for each stablished time, in order to avoid the effect of volume variation on the capacity of the bioadsorbent. These models include the pseudo-first-order [39] and pseudo-second-order [40]; as well as the Chien-Clayton model proposed by Chien and Clayton [41] and the intraparticle diffusion model proposed by Weber and Morris [42]. 

## 4. Conclusions

Based on the results described in this work it can be concluded that calcium alginate hydrogel beads (CAB) can be considered a bioadsorbent with a high capacity to remove from electroplating wastewater an elevated load of CN_f_ and other pollutants (Ni, Cu and Zn). The encapsulation of activated carbon or bio-oxidate grape marc in CAB only provided a slight enhance of removal efficiencies, therefore we propose the use of calcium alginate hydrogel by itself as bioadsorbent. The great availability, biodegradability and possibility to obtain calcium alginate biopolymers from secondary raw materials makes CAB an attractive alternative to obtain more biodegradable and eco-friendly adsorbents for the removal of hazardous residues. It was proved that the removal efficiency of CAB was increased when increasing the amount of bioadsorbent, being the best option a ratio 1.5:1 (*v*/*v*) between bioadsorbent/raw wastewater. The use of a second treatment cycle (ratio 1:1 *v*/*v*) allowed to reach removal efficiencies of 85.02% for CN_f_ and between 93.40–98.21% for the heavy metals evaluated, regarding the raw wastewater highly polluted. The adsorption equilibrium was reached after 30 min with adsorption capacities about 1177, 107.3, 39.5 and 1.52 mg/g for CN_f_, Ni, Cu and Zn, respectively, observing a high affinity for cyanide ions regarding heavy meals. Pseudo-second-order was the model that provided a better adjustment between theoretical and experimental data, observing a faster sorption rate and capacity for CN_f_ and Ni regarding Cu and Zn. The values for intraparticle diffusion rate constant suggest that cyanide ions diffuse more quickly than heavy metals between the particles of the adsorbent, playing film diffusion adsorption an important role. 

## Figures and Tables

**Table 1:** Composition of the untreated wastewater studied, from an electroplating industry, and the maximum allowable values for limited discharges into the public wastewater treatment plants.

Pollutant	Raw Wastewater (mg/L)	Maximum Values in Discharges (mg/L) ^b^	Maximum Values in Discharges (mg/L) ^c^
CN_f_	51,190 ± 415	0.50	-
CN_Total_	-	1.0	1.0
Ni	4899 ± 13	2.00	2.6
Cu	1904 ± 6	3.00	2.7
Zn	58.8 ± 0.5	2.00	2.6
Pb	N.D. ^a^	1.00	0.4
Cr (VI)	N.D. ^a^	0.5	-
Cr (Total)	N.D. ^a^	2.00	4.00
pH	12.45 ± 0.1	5.5–9	7.5–10

^a^ Not detected. ^b^ Decree 141/2012 about Public Service of Sanitation and Wastewater Treatment of Galicia, Official Journal of Galicia, 2012, N° 129, 26924-26958 [7]. ^c^ USEPA (United States Environmental Protection Agency), Electroplating and Metal Finishing Point Source Categories, Effluent Limitations Guidelines, Pretreatment Standards and New Source Performance Standards, 40 CFR, Part 413, 1983, 32462–32488 [6].

**Table 2:** Statistical analysis (ANOVA and *t*-test) for comparing the removal percentages obtained, for all pollutants, using the three bioadsorbents tested (CAB, EBGM and EAC).

	ANOVA		*t*-Test
	F Value ^a^	*p* Value	*t* Value ^b^
CN_f_	86.135	<0.001	2.585
Ni	4412.065	<0.001	10.11
Cu	416.796	<0.001	15.05
Zn	416.796	<0.001	1.532

^a^ F_critical_ = 5.143, ^b^ *t*_critical_ (*p* = 0.05) = 2.776; *t*_critical_ (*p* = 0.01) = 4.604.

**Table 3:** Percentages of removal of cyanide and heavy metals after two successive treatment cycles with calcium alginate hydrogel beads (CAB). The first cycle was carried out using a ratio (1.5:1 *v*/*v*) between bioadsorbent/raw wastewater and in the second cycle were used two different ratios (0.5:1 and 1:1 *v*/*v*).

**First Treatment Cycle (Ratio 1.5:1)**			
	**Raw Water (mg/L)**	**Water after** **1st Cycle (mg/L)**	**Removal (%)**
CN_f_	51,190 ± 415	15,659 ± 105	69.41 ± 0.21
Ni	4899 ± 13	1641 ± 5	66.50 ± 0.14
Cu	1904 ± 6	702 ± 2	63.13 ± 0.15
Zn	58.8 ± 0.5	13.2 ± 0.1	77.55 ± 0.24
**Second Treatment Cycle Ratio 0.5:1 (*v*/*v*)**			
		**Removal (%)**	
	**Water after** **2nd Cycle (mg/L)**	**Regarding** **Pre-Treated Water ^a^**	**Regarding Raw Water**
CN_f_	10,913 ± 79	30.31 ± 0.51 ^b^	78.68 ± 0.15 ^c^
Ni	105.3 ± 0.9	93.59 ± 0.12 ^b^	97.85 ± 0.04 ^c^
Cu	52.4 ± 0.5	92.53 ± 0.21 ^b^	97.25 ± 0.08 ^c^
Zn	6.01 ± 0.07	54.44 ± 0.64 ^b^	89.81 ± 0.14 ^c^
**Second Treatment Cycle Ratio 1:1 (*v*/*v*)**			
		**Removal (%)**	
	**Water after** **2nd Cycle (mg/L)**	**Regarding** **Pre-Treated Water ^a^**	**Regarding Raw Water**
CN_f_	7667 ± 79	51.04± 0.50 ^b^	85.02 ± 0.15 ^c^
Ni	87.5 ± 0.5	94.67 ± 0.04 ^b^	98.21 ± 0.01 ^c^
Cu	38.7 ± 0.4	94.49 ± 0.05 ^b^	97.97 ± 0.02 ^c^
Zn	3.90 ± 0.02	70.46 ± 0.16 ^b^	93.40 ± 0.04 ^c^

^a^ Values calculated respect to the pollutant contents found in the first-cycle treated wastewater. ^b,c^ Values statistically compared by means of *t*-test (*p* = 0.05) and significant differences were found between them.

**Table 4:** Kinetic parameters obtained after the adjustment of experimental data to the different kinetic models.

**Pseudo-First-Order**				
	**k_1_ (1/min)**	**q_e_ Exp (mg/g)**	**q_e_ Calc (mg/g)**	**r^2^**
CN_f_	0.0960	1177	160.1	0.9104
Ni	0.1018	107.3	13.83	0.9353
Cu	0.1326	39.5	10.44	0.9264
Zn	0.1108	1.52	0.415	0.9615
**Pseudo-Second-Order**				
CN_f_	0.00128	1177	1250	0.9999
Ni	0.01881	107.3	108.7	0.9999
Cu	0.03280	39.5	40.16	0.9999
Zn	0.65082	1.52	1.55	0.9998
**Chien-Clayton**				
	**α (min mg/g)**	**β (g/mg)**	**r^2^**	**α (min mg/g)**
CN_f_	5.43 × 10^11^	0.023	0.9421	5.43 × 10^11^
Ni	1.58 × 10^11^	0.261	0.9632	1.58 × 10^11^
Cu	2.66 × 10^5^	0.377	0.9969	2.66 × 10^5^
Zn	1247.3	8.382	0.9961	1247.3
**Intraparticle Diffusion**				
	**k_p_ (mg/g min^0.5^)**	**C (mg/g)**	**r^2^**	**k_p_ (mg/g min^0.5^)**
CN_f_	27.487	1024.9	0.9903	27.487
Ni	2.385	94.35	0.9958	2.385
Cu	1.609	31.11	0.9832	1.609
Zn	0.0727	1.130	0.9870	0.0727
